# The Many Faces of Protease-Activated Receptor 2 in Kidney Injury

**DOI:** 10.3390/biomedicines13020414

**Published:** 2025-02-08

**Authors:** Yuji Oe, Tetsuhiro Tanaka, Nobuyuki Takahashi

**Affiliations:** 1 Department of Nephrology, Tohoku University Graduate School of Medicine, Sendai 980-8574, Japan; 2 Division of Clinical Pharmacology and Therapeutics, Tohoku University Graduate School of Pharmaceutical Sciences & Faculty of Pharmaceutical Sciences, Sendai 980-0845, Japan

**Keywords:** coagulation proteases, chronic kidney disease, acute kidney injury, inflammation, fibrosis

## Abstract

Protease-activated receptor 2 (PAR2) is a seven-transmembrane, G-protein-coupled receptor that is activated by coagulation proteases such as factor VIIa and factor Xa and other serine proteases. It is a potential therapeutic target for kidney injury, as it enhances inflammatory and fibrotic responses via the nuclear factor-kappa B and mitogen-activated protein kinase cascades. The body of knowledge regarding the role of PAR2 in kidney disease is currently growing, and its role in various kidney disease models, such as acute kidney injury, renal fibrosis, diabetic kidney disease, aging, and thrombotic microangiopathy, has been reported. Here, we review the literature to better understand the various aspects of PAR2 in kidney disease.

## 1. Introduction

Protease-activated receptors (PARs) are four members of the G-protein-coupled receptor superfamily (PAR1-4). The N-terminus of PAR undergoes protease cleavage, and the tethered ligand activates itself [1,2,3]. Each PAR is activated by a specific protease, including coagulation proteases. The tissue factor (TF)–factor VIIa (FVIIa) complex activates PAR2, and the TF-FVIIa–factor Xa (FXa) complex activates PAR1 and PAR2, respectively. Thrombin targets PAR1, PAR3, and PAR4. Additionally, other serine proteases, including trypsin, tryptase, and matriptase, are also involved in PAR activation [1,2,3].

Tissue-factor-dependent coagulation is associated with numerous kidney diseases, including acute kidney injury (AKI) and chronic kidney disease (CKD), suggesting that coagulation proteases are upregulated in these conditions [4,5,6,7,8]. Furthermore, PARs are known to increase the inflammatory response and cause organ injury [9,10,11,12]. Therefore, PARs are attracting attention as a therapeutic target for kidney diseases.

To date, the basic evidence for the role of PAR2 in kidney disease has been accumulating. Harmful effects of PAR2 have been identified in some kidney injury models, including AKI, CKD, aging, and glomerulonephritis. Conversely, reno-protective effects of PAR2 have also been reported in other kidney injury models. There are excellent reviews of PARs and kidney disease [11,13], so here, we focus only on PAR2 and review in more detail the pathophysiology of PAR2, its role in models of kidney injury, and recent findings.

## 2. Protease-Activated Receptor 2

PAR2 is a seven-transmembrane G-protein-coupled receptor and one of the four PAR family members that was cloned from a mouse genome library in 1994 and from a human library in 1995 [14,15]. The human PAR2 molecule consists of 397 amino acids and contains an extracellular region (N-terminal and extracellular loop), a transmembrane region formed by seven pseudo-parallel helical sequences that are linked by three extra- and intracellular loops, and an intracellular C-terminus [2,16]. The activation of PAR2 involves proteolytic cleavage of the N-terminal region of R^36^/S^37^, exposing the tether ligand sequence S^37^LIGKV. This tether ligand interacts with its own extracellular loop and initiates intracellular signaling [15,17].

Well-known PAR2 activators include coagulation proteases. Among them, the TF-FVIIa complex and the TF-FVIIa-FXa complex target PAR2. Additionally, serine proteases, including trypsin, tryptase, and matriptase, can activate PAR2 [1,10,18]. Furthermore, cathepsin S, a cysteine protease, cleaves the N-terminus of PAR2 at a site that is distinct (G^40^/K^41^) from that of serine proteases to activate signaling [1,19].

PAR2 exhibits diverse signaling pathways in various pathophysiological conditions through different G protein α subunits such as Gα_q_, Gα_12/13_, and Gα_i_. Briefly, Gα_q_-mediated phospholipase C-beta activates downstream diacyl glycerol or inositol 1,4,5-triphosphate, resulting in Ca^2+^ release and protein kinase C activation. Gα_12/13_ increases Rho-Kinase activity. Gαi can regulate cAMP [3,10,20,21]. Downstream of these pathways, nuclear factor-κB (NF-κB) or mitogen-activated protein kinases (MAPKs) are activated, leading to PAR2-mediated inflammation, proliferation, and angiogenesis [10,22,23]. In addition, PAR2 can activate NLR-family pyrin-domain-containing 3 inflammasomes and promote vascular dysfunction [24]. Furthermore, through the G-protein-dependent pathway, β-arrestins can conjugate with PAR2 as a scaffold protein, activate the extracellular-signal-regulated kinase (ERK) pathway, and initiate degradation and receptor recovery [10,25,26,27,28].

## 3. Expression of PAR2 in the Kidney

PARs are widely expressed in renal cells, but this expression is known to be species-specific. Regarding PAR2 expression, it is expressed in glomerular endothelial cells, mesangial cells, and renal tubular cells of human or mouse origin [4,29,30,31,32]. For podocytes, PAR2 is expressed in humans but is not predominantly expressed in mice [4,29]. Immunoreactive PAR2 was evident in glomerular mesangial cells and distal tubuli in normal rabbit kidneys [33]. PAR2 can be increased under disease conditions. Its expression in the whole kidney is elevated in diabetic nephropathy and renal fibrosis models, cisplatin-induced nephropathy, as well as adriamycin (ADR) nephropathy [34,35,36,37,38]. Moreover, the glomerular expression of PAR2 was elevated in a rat model of mesangial glomerulonephritis and diabetic db/db mice [39,40]. Immunohistochemistry and in situ hybridization studies have demonstrated that PAR2 expression levels are elevated in injured tubules in renal fibrosis models [37,41]. For humans, PAR2 mRNA levels were markedly elevated in renal biopsy samples from IgA nephropathy compared to the levels in the normal kidney. Furthermore, immunohistochemical staining indicated that PAR-2 was localized primarily in the proximal tubules and within the stromal infiltrate [42].

## 4. PAR2 Signaling in Renal Tubular Cells

**Figure 1:**
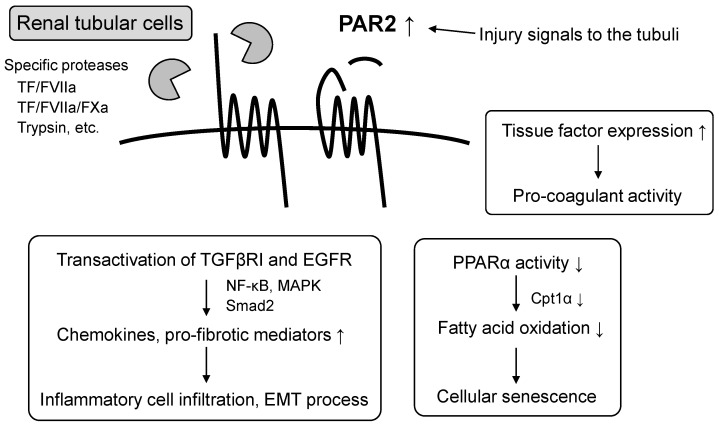
PAR2 signaling in renal tubular cells. PAR2 is increased by tubular injury and activated by specific proteases. PAR2 induces transactivation of both transforming growth factor-β receptor and epidermal growth factor receptor and activates transcription factors such as nuclear factor-kappa B (NF-κB), mitogen-activated protein kinase (MAPK), and Smad2, causing tubular inflammation and fibrosis. In addition, pro-aging and pro-coagulant effects of PAR2 have been demonstrated. TF/FVIIa, tissue factor–factor VIIa complex. TF/FVIIa/FXa, tissue factor–factor VIIa-factor Xa complex. TGFβRI, transforming growth factor (TGF)-β receptor 1. EGFR, epidermal growth factor receptor. EMT, epithelial–mesenchymal transition. PPARα, peroxisome proliferator-activated receptor α. Cpt1α, Carnitine palmitoyltransferase I α. ↑ up-regulated, ↓ down-regulated.

As noted above, PAR2 expression is abundant in tubular cells, and its expression is increased under disease conditions. Thus, PAR2-mediated signaling in tubular cells may be important in the pathogenesis of kidney injury. The proinflammatory and pro-fibrotic signaling functions of PAR2 were demonstrated using cultured human renal tubular cells [37,43,44,45]. The PAR2 agonist peptides SLIGKV and 2f-LIGRLO caused intracellular Ca^2+^ mobilization and induced secretion of proinflammatory and fibrotic cytokines such as tumor necrosis factor α, interleukin 6, plasminogen activator inhibitor 1 (PAI1), and connective tissue growth factor (CTGF) [44,45]. Transcription factors such as NF-κB, activator protein 1, and Smad2 were involved in these inductions [44]. Furthermore, signaling through PAR2-induced transactivation of both the transforming growth factor (TGF)-β receptor and epidermal growth factor receptor (EGFR), as well as PAR2-agonist-induced secretion of proinflammatory cytokines, was inhibited by TGF-β receptor-1 kinase inhibitor and EGFR kinase inhibitor treatments [44,45]. In another tubular epithelial cell line (NRK52E), PAR2 agonist peptide treatment increased the chemokine expression and promoted microphage migration via the MAPK signaling [37]. MAPK inhibitors also significantly decreased PAR2-induced markers of epithelial–mesenchymal transition (EMT) [37]. Moreover, the PAR2 agonist suppressed peroxisome proliferator-activated receptor α (PPARα) activity and carnitine palmitoyltransferase I α (Cpt1α) expression, which promotes cellular senescence [46]. The distinct role of PAR2 in pro-coagulant activity in human renal tubular cells was also addressed. PAR2 agonist peptides induce the synthesis and secretion of TFs that promote blood clotting in the cells, suggesting a local pro-coagulant effect of PAR2 in the kidneys [47]. Furthermore, TF synthesis induced by PAR2 was enhanced by high concentrations of glucose and inhibited by glycolysis and glycosylation [48] (Figure 1).

## 5. Regulation of Renal Hemodynamics and Ion Transport by PAR2

PAR2 contributes to vasorelaxation and regulates the blood pressure and renal hemodynamics [49,50,51]. Using isolated perfused rat kidneys, the activation of PAR1 caused renal vasoconstriction and markedly reduced the glomerular filtration rate. In contrast, the activation of PAR2 caused vasodilation and partially reversed the vasoconstriction that had been induced by angiotensin II. Moreover, PAR2-mediated vasodilation was partially inhibited by an inhibitor of nitric oxide (NO) synthesis, suggesting an endothelial nitric oxide synthase (eNOS)-dependent mechanism [51]. Another mechanism of hemodynamic control by PAR2 is the release of renin. In the isolated perfused kidney model, thrombin or PAR1 agonist treatment reduced the perfusate flow and inhibited renin secretion rates, whereas the PAR2-activating peptide SLIGRL caused concentration-dependent increases in renin release through a nitric oxide-dependent mechanism [52]. An additional study demonstrated that renin release facilitated by PAR2 was evident under acute inflammatory conditions [53]. Finally, PAR2 stimulates fluid reabsorption by promoting sodium reabsorption in cortical collecting duct cells and prevents potassium secretion via ERK signaling, and this may be involved in the regulation of blood pressure by PAR2 [54].

## 6. Role of PAR2 in Models of Kidney Injury

### 6.1. Acute Kidney Injury

Ischemia–reperfusion (I/R) injury is a major cause of acute kidney injury (AKI) and is characterized by an upregulation of TF-dependent hypercoagulability [55,56,57]. Therefore, subsequent PAR2 activation may be involved in this renal injury. In a study demonstrating the effects of low levels of the tissue factor hirudin and genetic deletion of PAR1 and PAR2 on the murine I/R model, low levels of TF and PAR1 deficiency were protective against renal failure, histological damage, and mortality. However, interestingly, PAR2 deficiency exerted no therapeutic effect, suggesting that PAR2 is likely not a significant pathway in this model [55].

Another commonly used model of AKI is the cisplatin-induced nephrotoxicity model. Renal TF and PAR2 expressions are elevated in cisplatin-treated mice [36]. Moreover, treatment with fondaparinux, a synthetic selective inhibitor of FXa, reduced cisplatin-induced nephrotoxicity in mice, suggesting that downstream PAR2 is involved in the injury [58]. To clarify the direct role of PAR2 in this type of kidney injury, we demonstrated that PAR2 deficiency ameliorates increases in blood urea nitrogen and plasma creatinine by cisplatin. Acute tubular damage scores and inflammatory cell infiltration were also improved. Similarly, apoptotic markers were also suppressed by PAR2 deletion [36].

### 6.2. Renal Fibrosis

Unilateral ureteral obstruction (UUO), a model of experimental hydronephrosis, is one of the most commonly used animal models to identify new mechanisms and treatments underlying renal fibrosis [59]. A study using an UUO-induced renal fibrosis model demonstrated that a lack of PAR2 reduced renal tubular injury, fibrosis, and collagen synthesis on day 7. Additionally, fibrosis-promoting markers such as CTGF and TGFβ gene expression, α-smooth muscle actin protein, and phospho-Smad2 were decreased in the kidney [45]. In another study that examined the localization of PAR2 expression using the same UUO model, in situ hybridization identified increased PAR2 expression in tubular cells, suggesting elevated tubular PAR2 signaling in renal fibrosis. However, PAR2 deficiency did not ameliorate tubular damage, fibrosis, and inflammatory cell infiltration in this study [41].

In adenine-induced nephropathy, absorbed adenine is metabolized to 2,8-dihydroxyadenine, causing tubular damage and renal fibrosis [60]. In this model, the gene expression levels of PAR1, 2, and 4 in the kidney were increased by adenine treatment [34,37]. Furthermore, immunohistochemistry confirmed the elevated protein expression levels of PAR2 in their tubuli [37]. We have demonstrated that a lack of PAR2 attenuated histological damage and decreased the expression levels of inflammation, fibrosis, and oxidative stress markers in this model [34]. Another study demonstrated a PAR2-mediated epithelial–mesenchymal transition process in adenine-induced nephropathy [37]. These two studies support harmful effects of PAR2 in adenine-induced nephrotoxicity and renal fibrosis.

### 6.3. Diabetic Kidney Disease

In diabetic mellitus, hyperglycemia leads to microvascular complications. Inflammation, endothelial dysfunction, and increased coagulability correlate with each other and play an important role in the development of its vascular complications [61,62]. In particular, the role of eNOS in diabetic microvascular injury has been clarified. Diabetic mice lacking eNOS develop massive albuminuria, glomerulosclerosis, and arteriolar hyalinosis, similarly to human diabetic kidney disease (DKD) [63,64]. Additionally, the activity of tissue-factor-dependent coagulation was increased by a lack of eNOS in DKD [6,65]. Therefore, the coagulation–PAR pathway is likely involved in diabetic nephropathy. Coagulation factor Xa is one of the coagulation proteases that activate both PAR1 and PAR2. We elucidated the pathological roles of the factor Xa-PAR2 pathway in diabetic Akita mice lacking eNOS [35]. The expression levels of factor X and PAR2 were elevated in diabetic kidneys. Edoxaban, an oral factor Xa inhibitor, and PAR2 deficiency improved glomerulosclerosis and urinary albumin excretion, and this was accompanied by a decrease in renal-inflammation–fibrosis-related markers in this model [35]. In another study, we determined the effects of dual inhibition of PAR1 and PAR2 on DKD in the same model. PAR1 or PAR2 antagonist (E5555 or FSLLRY, respectively) alone reduced glomerulosclerosis in the kidneys of diabetic mice. Additionally, the dual blockade of PAR1 and PAR2 by E5555 + FSLLRY improved histological injury in an additive manner, including mesangial expansion, glomerular macrophage infiltration, and type IV collagen deposition. The expressions of inflammation- and fibrosis-related genes in the kidney were also markedly reduced [66].

The role of PAR2 in streptozotocin-induced diabetic nephropathy was also examined [67]. A lack of PAR2 resulted in reduced albuminuria compared to that of wild-type mice, but mesangial expansion and collagen deposition was rather increased [67]. PAR2 deficiency increased the expression of PAR1 and fibrosis-inducible PAI1, suggesting that compensatory PAR1-derived signals may elevate pro-fibrotic responses in this model of DKD.

Cathepsin S (Cat-S) is a cysteine protease that is associated with cardiovascular risk in CKD and the progression of DKD [68,69]. It is also recognized as an activator of PAR2 [19]. Culture studies revealed that Cat-S selectively impairs glomerular endothelial cell integrity and barrier function via PAR2 [70]. They also observed that only CD68(+) intrarenal monocytes expressed Cat-S mRNA. Cat-S or PAR2 inhibitors ameliorated albuminuria and glomerulosclerosis in type 2 diabetic *db/db* mice, as well as albumin leakage into the retina and other structural markers of diabetic retinopathy. These findings suggest that monocyte/macrophage-derived Cat-S activated PAR2 and facilitated microvascular complications in diabetes [70].

### 6.4. High-Fat-Induced Nephropathy

Hypercoagulability is increased under obesity, as evidenced by increases in PAI1, TF, and platelets [71,72]. Additionally, targeting TF and PAR2 alleviated adipose tissue inflammation and insulin resistance in a study of diet-induced obesity mice and rats [73,74]. Obesity is a major risk factor for CKD, causing direct lipotoxicity and chronic inflammation in renal cells due to free fatty acids [75,76]. Therefore, PAR2 signaling can be involved in the pathogenesis of high-fat-induced nephropathy [77]. High fat diet exposure for 16 weeks in mice caused increases in blood urea nitrogen levels, oxidative stress and inflammation, and renal tubular injury. The expression level of tubular PAR2 was also increased in the model. PAR2 deficiency significantly reduced the inflammatory gene expression and inflammatory cell infiltration, as well as extracellular matrix protein accumulation [77].

### 6.5. Aging Kidneys

The accumulation of senescent cells in tissues is associated with age-related diseases, such as CKD, and cellular senescence exacerbates CKD through the secretion of inflammatory and pro-fibrotic factors [78]. A recent study using single-cell RNA-seq technology identified elevated inflammatory and pro-coagulant microenvironments in aging glomerular endothelial cells that may contribute to age-related kidney injury [79]. A study demonstrated the relationship between PAR2 and aging kidneys. PAR2 activation induced cellular senescence and inhibited fatty acid oxidation in cultured tubular cells. An elevated PAR2 expression was observed in the renal tubular cells of aged rat kidneys. Furthermore, a lack of PAR2 alleviated age-related cellular senescence markers and fibrosis in 20-month-old mice. Thus, inhibiting PAR2 can be a new therapeutic option to prevent age-related kidney injury [46].

### 6.6. Hyperuricemic Nephropathy

Uric acid is the major metabolic end product of purine metabolism. It is excreted mainly through the kidneys, and hyperuricemia is commonly observed in patients with CKD [80]. Mechanistically, soluble and crystalline uric acids are inflammatory and have been demonstrated to amplify the inflammasome and NF-kB pathway, exacerbating renal injury [81,82]. A study has reported renal dysfunction, tubular inflammation, and upregulation of PAR2 in the kidney in a rat model of hyperuricemia induced by adenine and ethambutol. Hyperuricemic rats who were treated with AZ3451, a selective antagonist of PAR2, indicated that the blocking of PAR2 reduced the phosphatidylinositol 3-kinase (PI3K)/AKT/NF-κB pathway and histological severity. Therefore, PAR2 is a promising therapeutic target for hyperuricemia-induced inflammatory reactions [83].

### 6.7. Focal Segmental Glomerulosclerosis

Focal segmental glomerulosclerosis (FSGS) is a histologic pattern of glomerular injury, in which podocytes are predominantly injured. Patients with FSGS often present with nephrotic syndrome [84]. The expression level of PAR2 was increased in the kidney of ADR-induced nephropathy, a model of FSGS [38]. The inhibition of PAR2 signaling by FSLLRY-NH2 suppressed inflammation and improved renal function and glomerular injury. It also specifically restored nephrin expression and inhibited the enhancement of TGF-β1, caspase-9, and desmin. Moreover, PAR2 antagonist treatment restores nephrin and suppresses desmin and caspase 9 expression in cultured murine podocytes that are treated with TGF-β1 [38].

### 6.8. Experimental Glomerulonephritis

It has been reported that fibrin and other coagulation factors are increased in crescentic nephritis, exacerbating glomerular injury [85]. The role of PAR-2 in anti-glomerular basement membrane antibody-induced crescentic glomerulonephritis was examined in PAR2-deficient and wild-type mice [86]. PAR2 deficiency reduced glomerular crescent formation, proteinuria, serum creatinine, and fibrin deposition. Additionally, a lack of PAR2 decreased the expression of renal PAI1 and increased renal matrix metalloproteinase-9 activity. These results indicated that PAR2 promotes inflammation in crescentic glomerulonephritis [86]. Similarly, another study demonstrated that the pharmacological inhibition of PAR2 by GB88 inhibited glomerular crescent formation in nephrotoxic serum nephritis in rats [87]. This treatment also significantly reduced glomerular thrombosis and decreased macrophage infiltration into Bowman’s lumen. Conversely, other disease indicators, such as the degree of proteinuria, renal function, inflammation, and tubular damage, were not altered by GB88 [87].

### 6.9. Lupus Nephritis

Systemic lupus erythematosus (SLE) is an autoimmune disease, and lupus nephritis is one of its most severe organ manifestations [88]. PAR2 activators (coagulation proteases and cathepsin S) are upregulated under the pathogenesis of SLE [89,90]. The administration of a cathepsin S antagonist to MRL/lpr mice, a lupus-prone mouse, was demonstrated to suppress systemic autoimmunity and inflammation. Conversely, cathepsin S exacerbated glomerular endothelial injury and albuminuria in a PAR2-dependent manner [91]. Moreover, the novel potent PAR2 antagonist punicalagin significantly ameliorated kidney injury and splenomegaly and reduced the expression of intercellular adhesion molecule 1 (ICAM-1) and vascular adhesion molecule 1 (VCAM-1) in NZB/W F1 lupus mice [92]. These two studies support the injurious role of PAR2 in lupus nephritis. On the contrary, we have demonstrated that the administration of FSLLRY-NH2, a PAR2 antagonist, to MRL/lpr mice exacerbated inflammatory cell infiltration into the glomeruli and increased the inflammatory cytokine expression [93]. Studies on SLE mice lacking PAR2 may reveal a more precise role for PAR2 in lupus nephritis.

### 6.10. Kidney Allograft Rejection

Renal transplantation is an option for patients with end-stage kidney disease, but immunosuppressive therapy is needed to prevent acute allorejection [94]. Cat-S is involved in the maturation of major histocompatibility complex class II in antigen-presenting cells that may elicit alloantigen recognition and allograft rejection [95]. Additionally, Cat-S causes microvascular injury via PAR2 [70]. Thus, both Cat-S and PAR2 can be therapeutic targets in acute renal allograft rejection. A study demonstrated that Cat-S inhibitors significantly protected the graft from tubulitis and intimal arteritis and decreased the expression of proinflammatory mediators. Furthermore, PAR2 deficiency also partially protected the grafts from tubulitis and reduced the expression of inflammation-related genes, suggesting that the inhibition of the Cat-S-PAR2 pathway is a therapeutic option for acute renal allograft rejection [96].

### 6.11. Thrombotic Microangiopathy

Thrombotic microangiopathy (TMA) is characterized by glomerular fibrin deposition, endothelial damage, and thrombus formation in the glomerular microvasculature and subsequent renal damage [97]. TMA is caused by a wide range of diseases, including thrombotic thrombocytopenic purpura (TTP); hemolytic uremic syndrome (HUS); and preeclampsia, drug, and autoimmune diseases such as antiphospholipid syndrome [97]. A study demonstrated that the low expression of tissue factors reduced renal injury in a mouse antiphospholipid antibody (aPL)-induced TMA model [98]. Furthermore, in mice lacking the TF cytoplasmic domain or PAR2, neutrophil activation was ameliorated, and pregnancy outcomes were improved. This indicates that PAR2 plays an important role in the pathogenesis of aPL-induced preeclampsia [99]. Contrary to this concept, there are some reports indicating that PAR2 protects endothelial cells. The activation of PAR2 may induce the proliferation of endothelial cells expressing endothelial protective factors, including vascular endothelial growth factor (VEGF) and angiopoietins [100,101,102]. Moreover, pregnant PAR2-deficient mice exhibited suppressed placental angiogenesis and impaired fetal growth [103]. On the basis of these findings, PAR2 deficiency exacerbated albuminuria and glomerular endothelial injuries and reduced the expression levels of VEGF and angiogenesis-related chemokines in the kidney injury model combining eNOS deficiency with an anti-VEGF antibody. The suppression of the angiogenic response by PAR2 deficiency may explain the exacerbation of glomerular injuries in the model [104]. These results indicate that PAR2 may protect against VEGF-inhibitor-induced glomerular injury.

## 7. Conclusions and Future Perspectives

We reviewed several roles of PAR2 in kidney injury (Table 1). PAR2 activators in patients with kidney diseases are elevated, and numerous reports suggest that PAR2 induces inflammatory and pro-fibrotic responses that exacerbate kidney injury, thus making it a promising drug target. However, there is still no treatment for kidney disease that focuses on PAR2. The inhibition of coagulation factors that activate PAR2 may be an option. The anticoagulant danaparoid has been reported to decrease proteinuria in human DKD [105], and direct oral factor Xa inhibitors have been widely used in recent years to prevent thromboembolism in patients with atrial fibrillation. However, unnecessary coagulation inhibition increases the risk of bleeding [106,107]. Therefore, the development of specific PAR2 inhibitors is expected. Small-molecule compounds, peptides, and antibodies with PAR2-inhibitory activity continue to be developed, and the crystal structure of PAR2 has also been elucidated [108,109,110]. Interestingly, Phase 1 clinical trials with PAR2-neutralizing antibodies (MEDI0618) have been performed for chronic pain (NCT04198558). We expect that PAR2 inhibitors may be utilized for the treatment of inflammatory diseases, including kidney diseases, in the near future.

## Figures and Tables

**Table 1:** List of animal studies showing the role of PAR2 in kidney injury.

	Species	Intervention	Outcome	Ref
*Acute kidney injury*				
I/R injury	Mouse	*PAR2* *^−/−^*	No change in serum creatinine	[55]
Cisplatin-induced	Mouse	*PAR2* *^−/−^*	Plasma creatinine, tubular injury, and inflammation ↓	[36]
*Renal fibrosis*				
UUO	Mouse	*PAR2* *^−/−^*	Tubular injury, fibrosis, and CTGF expression ↓	[45]
UUO	Mouse	*PAR2* *^−/−^*	No change in tubular damage	[41]
Adenine-induced	Mouse	*PAR2* *^−/−^*	Tubular injury and cytokine expression ↓	[34]
Adenine-induced	Mouse	*PAR2* *^−/−^*	BUN, fibrosis, and EMT markers ↓	[37]
*Diabetic kidney disease*				
Akita mice; *eNOS^+/−^*	Mouse	*PAR2* *^−/−^*	Albuminuria, mesangial expansion, and inflammation ↓	[35]
Akita mice; *eNOS^+/−^*	Mouse	FSLLRY (PAR2 inhibitor) E5555(PAR1 inhibitor)	Improved mesangial expansion, macrophage infiltration, and collagen IV deposition by dual blockade of PAR1 and PAR2	[66]
Streptozotocin	Mouse	*PAR2* *^−/−^*	Albuminuria decreased, but glomerular injury deteriorated	[67]
db/db mice	Mouse	RO5461111 (Cat-S inhibitor) GB83 (PAR2 inhibitor)	Cat-S and PAR2 inhibitors alleviate glomerulosclerosis	[70]
*High-fat-induced nephropathy*				
High-fat diet for 16 weeks	Mouse	*PAR2* *^−/−^*	BUN, renal inflammation, and fibrosis ↓	[77]
*Aging kidney*				
20-month-old	Mouse	*PAR2* *^−/−^*	Renal fibrosis and senescence ↓; fatty acid oxidation ↑	[46]
*Hyperuricemic nephropathy*				
Adenine- and ethambutol-induced	Rat	AZ3451 (PAR2 inhibitor)	Tubular dilation, inflammation, and PI3K/AKT/NF-κB pathway ↓	[83]
*Focal segmental glomerulosclerosis*				
ADR-induced	Rat	FSLLRY (PAR2 inhibitor)	Serum creatinine, proteinuria, and inflammation ↓; nephrin ↑	[38]
*Experimental glomerulonephritis*				
Anti-GBM Ab-induced	Mouse	*PAR2* *^−/−^*	Serum creatinine, proteinuria, glomerular crescent formation ↓	[86]
Nephrotoxic serum-induced	Rat	GB88 (PAR2 inhibitor)	Glomerular crescent formation ↓; no change in renal function	[87]
*Lupus nephritis*				
MRL/lpr	Mouse	RO5459072 (Cat-S inhibitor) GB83 (PAR2 inhibitor)	Cat-S causes glomerular endothelial injury and albuminuria via PAR2.	[91]
MRL/lpr	Mouse	FSLLRY (PAR2 inhibitor)	Glomerular injury and inflammation are deteriorated	[93]
NZB/W F1	Mouse	Punicalagin (PAR2 inhibitor)	Kidney injury, proteinuria, and splenomegaly ↓	[92]
*Kidney allograft rejection*				
Kidney transplant model	Mouse	RO5461111 (Cat-S inhibitor) *PAR2-/-*	Tubulitis, intimal arteritis, and inflammation ↓	[96]
*Thrombotic microangiopathy*				
Anti-VEGF Ab; *eNOS^−/−^*	Mouse	*PAR2* *^−/−^*	Albuminuria, podocyte, and endothelial injuries are deteriorated	[104]

Abbreviations: PAR, protease-activated receptor; I/R, ischemia/reperfusion; UUO, unilateral ureteral obstruction; eNOS, endothelial nitric oxide synthase; ADR, adriamycin; GBM, glomerular basement membrane; Ab, antibody; VEGF, vascular endothelial growth factor; eNOS, endothelial nitric oxide synthase; CTGF, connective tissue growth factor; BUN, blood urea nitrogen; EMT, epithelial-to-mesenchymal transition; Cat-S, cathepsin S; PI3K, Phosphoinositide 3-kinase; NF-κB, nuclear factor-κB; ↑ up-regulated; ↓ down-regulated.
